# Sigma-2: Multiple sequence alignment of non-coding DNA via an evolutionary model

**DOI:** 10.1186/1471-2105-11-464

**Published:** 2010-09-16

**Authors:** Gayathri Jayaraman, Rahul Siddharthan

**Affiliations:** 1 The Institute of Mathematical Sciences, Taramani, Chennai 600 113, India

## Abstract

**Background:**

While most multiple sequence alignment programs expect that all or most of their input is known to be homologous, and penalise insertions and deletions, this is not a reasonable assumption for non-coding DNA, which is much less strongly conserved than protein-coding genes. Arguing that the goal of sequence alignment should be the detection of *homology *and not *similarity*, we incorporate an evolutionary model into a previously published multiple sequence alignment program for non-coding DNA, Sigma, as a sensitive likelihood-based way to assess the significance of alignments. Version 1 of Sigma was successful in eliminating spurious alignments but exhibited relatively poor sensitivity on synthetic data. Sigma 1 used a *p*-value (the probability under the "null hypothesis" of non-homology) to assess the significance of alignments, and, optionally, a background model that captured short-range genomic correlations. Sigma version 2, described here, retains these features, but calculates the *p*-value using a sophisticated evolutionary model that we describe here, and also allows for a transition matrix for different substitution rates from and to different nucleotides. Our evolutionary model takes separate account of mutation and fixation, and can be extended to allow for locally differing functional constraints on sequence.

**Results:**

We demonstrate that, on real and synthetic data, Sigma-2 significantly outperforms other programs in specificity to genuine homology (that is, it minimises alignment of spuriously similar regions that do not have a common ancestry) while it is now as sensitive as the best current programs.

**Conclusions:**

Comparing these results with an extrapolation of the best results from other available programs, we suggest that conservation rates in intergenic DNA are often significantly over-estimated. It is increasingly important to align non-coding DNA correctly, in regulatory genomics and in the context of whole-genome alignment, and Sigma-2 is an important step in that direction.

## Background

Evolutionary models seek to describe the process by which DNA changes over time, while sequence alignment is the computational task, given two or more sequences of DNA, of determining which stretches of nucleotides may have arisen from a common ancestor. It seems logical to combine these goals, and we present an attempt to do so here. We specifically address non-coding DNA of unknown function, but it is straightforward to include functional models of DNA (such as selection for protein-binding), and we hope in the future also to extend this approach to protein-coding DNA and amino-acid sequences.

The motivation for using an evolutionary model in multiple sequence alignment is this: rather than simply optimise the "similarity" of two sequences by some "metric", we want to assess which similarities are unlikely to have occurred by chance. In other words, given two sequences, or two sets of sequences, we want to know whether or not to align them, by estimating the likelihood of observing such sequences under two hypotheses: that they are related (under our evolutionary model, with an unknown common ancestor); or that they are not related.

This is less of an issue in aligning protein-coding genes, which tend to be rather well conserved. Non-coding DNA, however, can contain strongly conserved regions (such as regulatory regions) interspersed among weakly-conserved regions. In an extreme case, we recently found [1] that the centromeric regions in two closely-related yeast species have *no *detectable homology, even though the neighbouring ORFs are well-conserved and syntenous, and most intergenic regions are well-conserved too. It is important that a sequence alignment program, when used on non-coding DNA, be able to distinguish genuine ancestral relatedness from chance similarity.

We start with a quick review of several evolutionary models, but it is important to note the difference in motivation: while most previous researchers have been interested in estimating evolutionary distances and constructing phylogenetic trees based on observed substitution patterns, we are interested in using known or estimated evolutionary history to evaluate differing hypotheses relating to the evolution or function of individual subsequences. Specifically, we have recently, in the motif-finder PhyloGibbs, [2,3] used an evolutionary model, as described in Methods, to evaluate the competing hypotheses that short stretches of sequence may be "regulatory" or "background". Here we use a similar evolutionary model to perform multiple sequence alignment by evaluating the hypotheses that two (sets of) subsequences from two longer (sets of) sequences may, or may not, be homologous. The word "homology" is used, throughout, in the sense of "evolutionary relatedness" [4], and not merely "similarity". A principal goal of the alignment program described here is that alignments reported by it should indicate homology, to a high degree of confidence.

Most evolutionary models trace their lineage to the work of Jukes and Cantor [5]. Their model assumes neutral evolution, independent evolution of nucleotides and a uniform mutation rate from any nucleotide to any other. Improvements to that model have largely consisted of using realistic mutation matrices that take account of differing mutation rates between different nucleotides: in particular, the fact that transitions (purine-purine or pyrimidine-pyrimidine) are much more common than transversions (purine-pyrimidine or vice versa). Kimura [6,7] accounted for differences in transition and two types of transversion rates. Further work along these lines has been done by Tamura [8], Tamura and Nei [9] and others. The most general reversible model was described by Tavaré [10], and the general 12-parameter model was discussed by Rodríguez *et al*. [11]. Meanwhile, Felsenstein [12] introduced a model, that we discuss further below, where mutation rates represent equilibrium frequencies for nucleotides. Hasegawa *et al*. [13] amended this method to take account of differing frequencies of transition and transversion. Heterogeneity of sequence and differing rates of fixation at different loci have been considered by various authors, starting from Uzzell and Corbin [14]. We do not consider this problem in detail here, but our model can be modified to include prior knowledge of sequence function and heterogeneity of sequence composition.

One shortcoming of such models is that they do not explain some significant observed features of DNA, the most basic of which is the fact that nucleotides are correlated, not independent. If one considers abundances of neighbouring nucleotides (dinucleotides), they differ significantly from what would be expected from their individual frequencies: for example, AA is usually over-represented while CG is underrepresented (in vertebrates, CG is severely underrepresented because methylation of the C makes it likely to mutate to a T [15]). Attempts have been made to address this by various authors. Arndt and Hwa [16] use dinucleotide substitution matrices instead of single-nucleotide matrices. While sufficient to account for the most important effects, this assumes that the mutation of certain dinucleotides is preferred. Sometimes this is true (for example, the CG dinucleotide in vertebrates), but in other cases selection forces (some of which are discussed below) could well be operating. Also, such an approach still does not account for longer-ranged correlations in DNA, which exist to significant distances in non-coding DNA, as first noted by Peng *et al*. [17]. Baele *et al*. [18] observe complex substitution behaviour, and argue that incorporating context-dependent substitution effects is worthwhile.

We argue that, even in the absence of known function, mutating intergenic sequence can have a cost in fitness, and selection and fixation could be operating on large parts of the genome--perhaps the majority. In a recent study of centromeric DNA in two *Candida *species [1], we calculated a substitution rate of 27% between those species from synonymous codon substitutions; correcting this with known codon biases gave a substitution rate of 42%, which was our best estimate at a neutral rate. However, the substitution rate in conserved intergenic sequence is much lower than either of these estimates (about 17%). Meanwhile, the centromeres appear to have diverged much faster than our best neutral rate would suggest--implying either that the centromeres evolve neutrally while the rest of the genome is under significant selection pressure, or that centromeres evolve at a "faster than neutral" rate, or both. It is possible that structural and stability requirements, the necessity to bind nucleosomes [19], and other biophysical considerations constrain the evolution of DNA.

We recently used an evolutionary model, in the context of the motif finder PhyloGibbs [2,3], that represents the polar opposite of neutral evolution: it assumes that fixation of nucleotides after mutation is perfect--that is, the distribution of mutated nucleotides matches the distribution found in sites elsewhere of similar function (which may be very different from a genomic average distribution). A similar approach was used in the *cis*-regulatory module predictor Stubb [20]. This is in fact the model of Felsenstein [12], with a slightly different interpretation and a very different motivation. The model is reviewed in Methods, "Evolutionary Model". However, while it is important to consider fixation (especially in the motif-finding context), the assumption of perfect fixation may be extreme and unrealistic. We address that issue here, thereby connecting with other models from the literature: we have a model that resembles the "general reversible model", with the inclusion of fixation but not "perfect fixation".

We then use this evolutionary model to address the problem of sequence alignment: specifically, we use this model to calculate the log-likelihood ratio of sequences being related, to being unrelated. We modify our previously published multiple sequence alignment program Sigma [21] to use this as a scoring scheme. The key goal of Sigma is to minimise spurious alignments (that is, alignments of sequence that are not likely to be homologous), a significant issue in non-coding DNA, where highly conserved segments can be interspersed with long insertions and deletions. This was achieved by calculating the *p*-value for the score of each locally aligned region, that is, calculating the probability of observing such a score under the "null hypothesis" that the sequences are not ancestrally related: only matches with sufficiently low *p*-values are considered for alignment. While one other program that we are aware of, Dialign 2 [22], also used a *p*-value as a criterion, our calculation of the *p*-value is different in details, as described in Methods. We tested several programs in the earlier paper [21] and showed that they produce spurious alignments even for randomly-generated DNA, and show significant error rates in aligning synthetic sequence; while Sigma (version 1) was much less sensitive (that is, it aligned a smaller fraction of nucleotides compared to other programs), we showed that the motif finder PhyloGibbs [2] exhibited better performance when its input data was aligned with Sigma-1, suggesting that its alignments were biologically more realistic.

Sigma-2, the modification of Sigma-1 that features the evolutionary model described here, proves to be substantially more sensitive than Sigma-1 on synthetic data (its sensitivity is now comparable to other programs), while maintaining a very low error rate and refusing to align sequence that is not related. We demonstrate this on both synthetic and genomic (yeast) DNA. The results indicate the benefits of including selection and fixation in an evolutionary model, of basing the problem of multiple sequence alignment on such a model, and of comparing results with the "null model" of unrelatedness, and insisting on stringent *p*-values to report alignments.

Ours is not the first attempt to include evolutionary considerations in sequence alignment, but it differs in details. Thorne *et al*. [23,24] have previously considered including an evolutionary model in pairwise sequence alignment. Their main focus was the treatment of insertions and deletions. Steel and Hein [25] extended that approach to sequences on a tree. The focus of our work is different: we focus on gapless local alignments, assuming that non-coding DNA will contain large insertions and deletions which will be accounted for by assembling the gapless alignments; and rather than consider the overall "maximum likelihood" alignment, we insist on a stringent *p*-value for the log-likelihood-ratio that we calculate for each local alignment. Below we benchmark our program against ten other widely-used multiple sequence alignment programs.

## Results and Discussion

We performed three sets of benchmarks, on synthetic and real (yeast) data, comparing Sigma-2 with eleven other programs: the previous version of Sigma (version 1.1.3), DiAlign-TX version 1.0.2 [26], T-Coffee version 8.06 [27], ClustalW version 2.0.11 [28,29], KAlign version 2.04 [30], MLagan version 2.0 [31], Muscle version 3.7 [32], PCMA version 2.0 [33], FSA version 1.15.3 [34], Pecan version 0.8 [35], MAVID version 2.0 build 4 [36].

### Benchmark on yeast data: discriminativeness

While synthetic benchmarks are better quantifiable, real DNA exhibits complexities difficult to capture in synthetic data. Here we describe the performance of Sigma-2 and other programs on yeast data. "Reference alignments" being unavailable, we measure performance indirectly: we compare the alignments produced by various programs for orthologous DNA, with alignments by the same programs for non-orthologous DNA.

We used 947 genes for *Saccharomyces cerevisiae *for which there existed a kilobase of upstream intergenic (non-coding) sequence, and for which the orthologous genes in four other species (*S. paradoxus*, *S. mikatae*, *S. bayanus *and *S. kudriavzveii*) also had a kilobase of upstream non-coding sequence. Thus, the benchmark consisted of aligning 947 files, each containing 1000 bp of orthologous non-coding sequence. We also generated 947 "shuffled" files, that contained the same upstream sequences from the same five species in each file, but entirely *non*-orthologous: that is, each sequence in the original set was present in exactly one shuffled file, but no two sequences in a given shuffled file were orthologous. This was accomplished by ordering the genes and the species, and selecting upstream sequence from the *n *+ 100*k*(mod2)'th gene for the *k*'th species (*k *= 0, 1, 2, 3, 4).

While we cannot quantify the accuracy of alignment on the orthologous sequences, we can say with some confidence that very little sequence from the "shuffled" set is likely to be genuinely homologous; so a program whose alignments indicate homology rather than mere "similarity" should not report significant similarity in the second set of sequences. At a minimum, there should be significant gap in results on the two sets.

Table 1 reports the average number of aligned nucleotides per input nucleotide for each program and each data set. That is, it shows the total number of matches per nucleotide summed over all nucleotides, divided by the total number of nucleotides. Since there are five sequences of equal length in each set, the theoretical maximum for this number is 4. Sigma-2 detects a significantly greater degree of similarity in the "orthologous" files, and a lesser degree of similarity in the "shuffled files", than its predecessor, Sigma-1.1.3. Both versions report a little under two matches for each nucleotide in the orthologous files, and very few matches per nucleotide in the shuffled files. Of the remaining programs, only DiAlign-TX, FSA and Pecan report a significant gap in results in the two data sets. Some programs, in fact, produce significantly *more *alignment in the shuffled set than in the genuinely orthologous set (approaching, in fact, the theoretical maximum of 4): an odd result that throws doubt on the utility of those programs in alignment of non-coding DNA sequence.

**Table 1:** Performance in aligning yeast sequence

Program	dataset	**Matches per base**^***a***^	dataset	**Matches per base**^***a***^	Difference
Sigma-2	orthologous	1.9893	shuffled	0.0031	1.9862
Sigma-1.1.3	orthologous	1.8688	shuffled	0.0050	1.8638
FSA	orthologous	2.4695	shuffled	0.1465	2.3230
Dialign-TX	orthologous	2.7498	shuffled	0.4539	2.2959
Pecan	orthologous	3.0234	shuffled	0.4430	2.5804
Mavid	orthologous	3.3181	shuffled	2.8248	0.4933
T-Coffee	orthologous	3.5582	shuffled	3.3495	0.2487
Clustal-W	orthologous	3.6202	shuffled	3.7517	-0.1315
KAlign	orthologous	3.7480	shuffled	3.8434	-0.0954
MLagan	orthologous	3.2956	shuffled	2.7082	0.5874
Muscle	orthologous	3.4541	shuffled	3.1901	0.2670
PCMA	orthologous	3.4822	shuffled	2.8941	0.5881

Performance in the yeast benchmark, described in the text, of 12 programs (including 2 versions of Sigma). 947 genes were selected, each of which had 1000 bp of non-coding upstream sequence in *S. cerevisiae *and four other species. Each upstream sequence and its four orthologues were aligned (dataset "orthologous"). In addition, 947 "scrambled" files were prepared each of which contained sequence from each of the five species, including no orthologous sequences, and these were aligned (dataset "scrambled"). "Matches per base" indicates the average number of nucleotides in other species that each nucleotide in the input data was aligned with (so its theoretical maximum is four). The difference between the "orthologous" and "scrambled" numbers is a measure of how discriminative the program is to genuine orthology.

All programs were run with their default command lines, except as follows: for Sigma-2, a file providing background dinucleotide frequencies, and another file providing transition rates, both files derived from yeast, were supplied. For Sigma-1.1.3, only the background file was supplied. DiAlign-TX was run with the parameter -12, the most stringent (and least sensitive) mode. FSA was run with the parameter --gapfactor 5, which increases its specificity. Mavid was run using the bundled perl script to automatically generate the phylogenetic tree. Pecan was fed the phylogenetic tree (((S. cer, (S. par, S. mik), S. kud), S. bay)).

Table 1 reports the most stringent options that we used for each program. In Table 2, we compare the effect of parameter changes in Sigma-2, Dialign-TX and FSA. In Sigma-2, we removed one or both of the background model option and the transition matrix option, resulting in assumptions of uniform nucleotide frequencies and/or uniform transition probabilities. It appears that assuming uniform background frequencies increases the number of erroneous alignments (in shuffled sequence) by a factor of more than 4, but slightly increases the number of alignments in orthologous sequence. Assuming uniform transition rates (with a realistic background model) hurts performance in both data sets. Making both the background and the transitions uniform causes a nearly tenfold increase in the alignments for shuffled sequence. If the threshold *p*-value for local alignments is increased from the default 0.002 to 0.2, and the background model and transition matrix are made uniform, then the alignment rate in orthologous sequence exceeds 2.5, while the alignment rate in non-orthologous sequence is about 0.06, still substantially less than all other programs.

**Table 2:** Yeast benchmark: effect of parameters

Program	dataset	**Matches per base**^***a***^	dataset	**Matches per base**^***a***^	Difference
Sigma-2 (defaults)	orthologous	1.9893	shuffled	0.0031	1.9862
Sigma-2 (no bg model)	orthologous	2.1319	shuffled	0.0133	2.1186
Sigma-2(no tr mat)	orthologous	1.9115	shuffled	0.0046	1.9069
Sigma-2(no bg, no tr)	orthologous	2.3799	shuffled	0.0275	2.3524
Sigma-2(*p *0.2)	orthologous	2.4139	shuffled	0.0389	2.3750
Sigma-2(*p *0.2, no bg, no tr)	orthologous	2.5615	shuffled	0.0586	2.5029
FSA (defaults)	orthologous	2.7996	shuffled	0.3572	2.4424
FSA (gap5)	orthologous	2.4695	shuffled	0.1465	2.3230
Dialign-TX (defaults)	orthologous	2.9501	shuffled	0.8576	2.0925
Dialign-TX (-l 2)	orthologous	2.7498	shuffled	0.4539	2.2959
Mavid (auto)	orthologous	3.3181	shuffled	2.8248	0.4933
Mavid (yeast tree)	orthologous	3.3393	shuffled	2.8713	0.4680

Performance on the yeast benchmark of a subset of the programs in Table 1 when command-line parameters are varied. In Sigma, "no bg" indicates a background model where each nucleotide is equally probable; "no tr" indicates a "uniform" transition matrix where a nucleotide can mutate to any other nucleotide with equal probability. In FSA, "gap5" indicates the command line option --gapfactor 5. In Dialign, -1 2 is the most stringent setting. Mavid tree options are as described in the text.

Of other programs, FSA and Dialign-TX still show substantial gaps between orthologous and shuffled sequence sets when run with their default settings; however, at their most stringent settings, both align much more shuffled sequence than Sigma-2 does at the least stringent setting tested above. Mavid was run with a tree corresponding to the yeast alignments, but the results did not greatly differ from the automatically-generated tree.

If the most basic task of a sequence alignment program is to distinguish homologous and non-homologous sequence, it seems that all but a few programs fail badly at that task, and Sigma-2 is by far the most stringent in rejecting non-homologous sequence.

Finally, one can ask: even in the alignment of orthologous sequence, to what extent do various programs agree with one another? We consider four programs that perform the most discriminative alignments in Table 1, namely Sigma 2, FSA, DiAlign-TX and Pecan. In the orthologous set, 4714791 pairs of nucleotides in total were identified by Sigma-2 as orthologous. Of these, 3889882 were identified by FSA, 3995809 by DiAlign-TX and 4022407 by Pecan. In other words, nearly 20% of the nucleotide pairs aligned by Sigma were *not *aligned by the other programs. We then ask, what about the alignments made by other programs and *not *by Sigma-2? 2073056 pairs of nucleotides are aligned by FSA and not by Sigma. Of these, 1829902 are also aligned by Pecan, but only 1465189 by DiAlign-TX. Meanwhile, Dialign-TX aligns many nucleotides that are omitted by Sigma-2 and FSA, and Pecan aligns many nucleotides that are omitted by all three programs. This level of disagreement, in a task of aligning five closely-related yeast species, indicates the difficulty of underlining non-coding DNA and the desirability of a conservative approach.

### Motif-finding benchmark on yeast data

To test our motif-finder PhyloGibbs [2] and PhyloGibbs-MP [3], we benchmarked its ability to identify transcription factor binding sites in yeast from the SCPD database [37]. Conversely, in the previous paper on Sigma [21], we measured the performance of PhyloGibbs 1.0 in detecting binding sites using sequence alignments generated from various programs. We repeat that benchmark here, using PhyloGibbs-MP. The reason to use SCPD is that it is a large database of experimentally validated binding sites. So measuring the performance of a motif finder in detecting these sites is an objective measure of its performance in the real world. While this benchmark does not directly measure the quality of the alignment, it is hoped that a more "correct" alignment will improve the performance of a motif-finder. We use a recently retrieved version of the SCPD database, after filtering out sites smaller than 3 bp. We were left with 512 sites upstream of 205 genes. Up to 1000 bp (or upto the next coding region, whichever was smaller) was extracted for each gene in *S. cerevisiae *and its orthologues from *S. paradoxus*, *S. bayanus*, *S. mikatae *and *S. kudriavzveii*. These were aligned using each of the alignment programs studied here, PhyloGibbs-MP was run on the aligned files individually (with a motif width of 10 bp, a predicted "site density" of 0.01 and "number of motifs" 3 for each file), and its site predictions compared with the annotated sites. Since the SCPD sites vary greatly in length (and, in addition, come from a variety of experimental methods), while our assumed motif width was 10 bp, an overlap of a single basepair was counted as a "hit".

**Figure 1:**
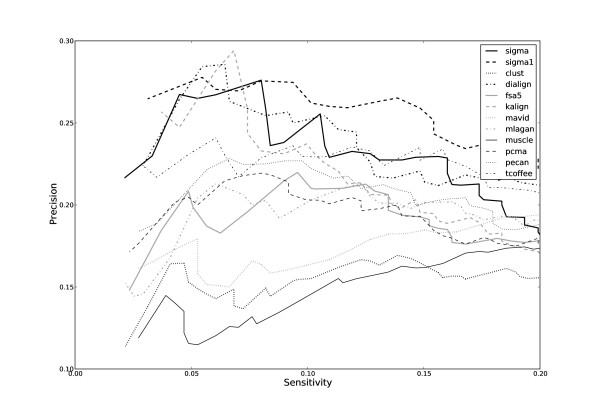
**Performance on site prediction using PhyloGibbs-MP**. The performance of the motif-finder PhyloGibbs-MP when various programs are used to align its input data. Upstream sequence for 205 genes from *S. cerevisiae *and up to 4 orthologues are aligned, and PhyloGibbs-MP is run on those alignments. Predictions are compared with known regulatory sites from the SCPD database. The figure shows the fraction of PhyloGibbs-MP predictions that overlap with SCPD sites ("precision") as a function of the fraction of SCPD sites retrieved by PhyloGibbs-MP ("sensitivity"). Sensitivity is varied by changing the cutoff for PhyloGibbs-MP's significance assessment.

The results are plotted in Figure 1, which shows the "precision" of PhyloGibbs-MP's predictions (the fraction of predictions that agree with SCPD) as a function of "sensitivity" (the fraction of SCPD sites that were found by PhyloGibbs-MP). The sensitivity is varied by changing the "cutoff" for the significance score reported by PhyloGibbs-MP. While not too many conclusions should be drawn from this limited benchmark. both versions of Sigma perform well over most of the sensitivity range, as does DiAlign-TX. Other good performers are Kalign and T-coffee. With several alignment programs, however, the motif-finding performance of PhyloGibbs-MP is surprisingly poor. Meanwhile, Sigma-1 mostly seems to fare better than Sigma-2: our hypothesis is that, though it is less sensitive in alignments than Sigma-2, it performs well in aligning functional binding sites (since these are probably better conserved) and this, in turn, helps bias PhyloGibbs-MP towards those sites (since the scoring in PhyloGibbs-MP rewards conserved sites). Perhaps this argument also helps explain the better performance of Sigma-2 compared to most other programs; but it does not explain the poor performance of FSA and Pecan. We cannot directly conclude from this benchmark that Sigma's alignments are more "correct" than others, but we can view it as supporting the use of Sigma in real-world applications where the correctness of the alignment is important.

### Benchmark on synthetic data

We generated sets of synthetic DNA that conformed to the evolutionary model described above, where each set was evolved from a 500 bp ancestral sequence and contained five descendants, each descendant sequence had a proximity *q *to the ancestor, and substitutions from the ancestor were made according to equation 9, with dinucleotide frequencies and an inverse substitution matrix estimated from yeast data. Values of *q *from 0.10 to 0.80, in increments of 0.05, were considered. In addition, insertions and deletions of short stretches of sequence (from 1 to 200 bp) were made with a small probability (0.02): in other words, around 10 insertions or deletions were expected per sequence. Each insertion and deletion applied only to a single descendant sequence (since each sequence was assumed to be independently evolved from the common ancestor). For each value of *q*, 100 independent sets of 5 sequences each were generated. This method of generating sequences also gave us the theoretical "correct" reference alignment for each set of sequences. Alignments were assessed on sensitivity to the reference alignments (that is, the fraction of aligned nucleotide pairs that were aligned in the program's output), but also on the error rate (the ratio of *incorrectly *aligned nucleotide pairs to the total number of aligned nucleotide pairs in the reference alignment) and the precision (the fraction of nucleotide pairs reported aligned that are aligned in the reference alignment). That is, if there are *N*_ref _aligned nucleotide pairs in the reference alignment, *N*_correct _aligned pairs in the reported alignment that are also aligned in the reference alignment, and *N*_incorrect _aligned pairs in the reported alignment that are *not *aligned in the reference alignment, we define

(1) $$\text{Sensitivity}=\frac{N_{\text{correct}}}{N_{\text{ref}}}$$

(2) $$\text{Error rate}=\frac{N_{\text{incorrect}}}{N_{\text{ref}}}$$

(3) $$\text{Precision}=\frac{N_{\text{correct}}}{N_{\text{correct}}+N_{\text{incorrect}}}$$

**Figure 2:**
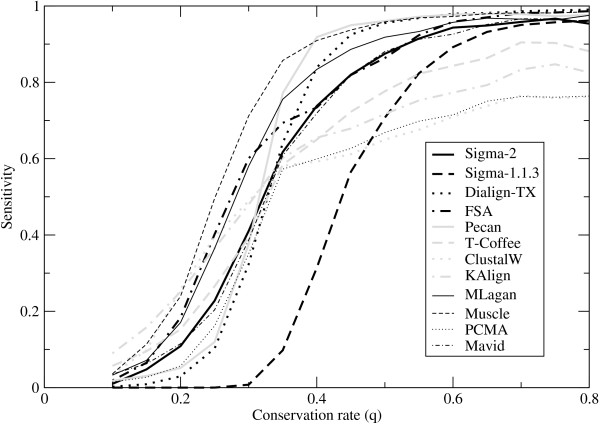
**Sensitivity on synthetic data**. The fraction of aligned nucleotide pairs in the reference alignment that are correctly reported by various programs ("sensitivity"), as a function of the conservation rate *q *between the sequences and their common ancestor.

**Figure 3:**
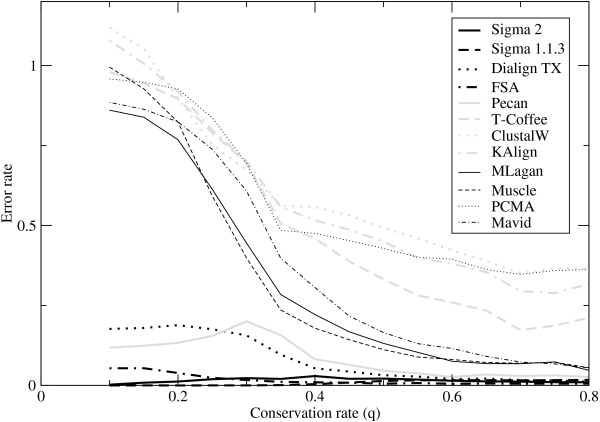
**Error rates on synthetic data**. The ratio of the number of aligned nucleotide pairs that are *incorrect *compared to the reference alignments, as a fraction of the total number of aligned nucleotide pairs in the reference alignments; plotted as a function function of the conservation rate *q *between the sequences and their common ancestor. Notably, this is more than 1 for some programs at low *q*: this means the number of incorrect alignments that they make exceeds the number of correct alignments in the reference alignment.

**Figure 4:**
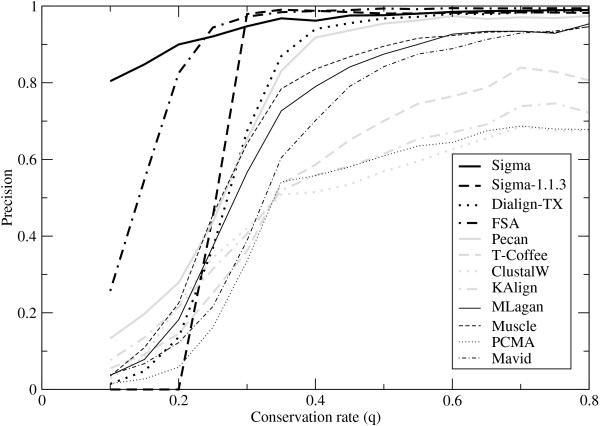
**Precision on synthetic data**. The fraction of aligned nucleotide pairs in the output alignments that are correctly aligned according to the reference alignment, as a function of the conservation rate *q*.

Figure 2 shows the sensitivity, Figure 3 shows the error rate, and Figure 4 shows the precision. Like its predecessor Sigma-1.1.3, Sigma-2 shows a very low error rate, but is much more sensitive, and comparable with the better performers in this aspect. The error rates in Figure 3 show a striking separation of Sigma (both versions), Dialign-TX, FSA and Pecan from the other programs. The precision data in Figure 4 show Sigma-2 outperforming all programs by far for weakly-conserved sequence (low *q*), and FSA somewhat outperforming it for intermediate conservation rates. For highly conserved sequence (*q >*0.5), Sigma (both versions), FSA, DiAlign-TX and Pecan show precisions close to 1; Muscle, MLagan and Mavid do just a little worse; and there is a substantial gap to the other programs.

## Conclusions

Benchmarking on synthetic data is of limited benefit in analysing real-world performance, but it is quantifiable. Kim and Sinha [38] recently did an exhaustive benchmark of six programs, and claim that their method generates data that "truly represent the variability observed in genomic data in terms of the difficulty of the alignment task". They observe degradation in performance with insertions, which is probably attributable to our observation that most programs spuriously align non-homologous sequence. They also observe that Pecan is not susceptible to this problem and that its performance was superior to all other programs, in agreement with what we see in yeast data (they did not benchmark FSA or Sigma-1), but in contrast with our observation in these synthetic data benchmarks. This supports their claim that their generated data are biologically realistic.

However, our "homology discrimination" benchmarks on yeast data are, we believe, of greater interest because of their simplicity and the somewhat unexpected results. Arguably the goal of sequence alignment should be to detect *homology *and not *similarity*, since the former is a well-defined biological concept meaning "having a common ancestry" [4] and the latter is not always unambiguous or even meaningful. We argue further that a sequence alignment program should err on the side of caution, that is, though it may fail in some cases to detect genuine homology, it should not incorrectly claim homology where none exists. Other than Sigma-2, all programs tested here fail, in differing degrees, on this criterion. The most effective at rejecting spurious alignments is FSA with a stringent gap factor. The only other programs that strongly distinguish the homologous sequences from the shuffled sequences are Pecan and Dialign-TX, but they still spuriously predict a homologous nucleotide for half, or more, of the nucleotides in the shuffled set. This performance, meanwhile, is far superior to all the other programs tested, which predict over two homologues per nucleotide in the shuffled set, and in some cases predict *more *homology in the shuffled set than in the genuinely homologous set. We feel therefore that these programs should *not *be used to align non-coding DNA (which was, in any case, not their primary purpose). This is particularly important since it is increasingly important to align, not just non-coding DNA, but whole genomes, and some of the programs described here have been used for that task; and the error rates seen here on the shuffled yeast data are a matter of concern.

With the default settings of Sigma-2 (which cause it to predict only 0.003 homologues per nucleotide in shuffled sequence), it predicts just under 2 homologues per nucleotide in orthologous sequence. With the loosest settings that we tested--a *p*-value of 0.2 for alignment, uniform background model, uniform transition matrix--Sigma-2 predicts close to 0.06 homologues per nucleotide in shuffled sequence (an error rate nearly 20 times larger) and over 2.5 homologues per nucleotide in non-shuffled sequence. FSA, run with a gapfactor of 5, performs worse on both counts: it predicts fewer homologues in the orthologous set and more homologues in the shuffled set. Other programs predict more homologues in both sets. Based on the predictions by Sigma-2, FSA, Pecan and Dialign-TX, we estimate that the true conservation rate between these species is probably around 2.5 homologues per nucleotide, and the significantly higher predictions of the other programs are unreliable. This is probably because of the abundance of insertions and deletions in intergenic sequence.

Sigma-1 was originally designed to reject such spurious alignments, and benchmarks on synthetic and real data showed that it performed well on this criterion, but was also less sensitive than other programs in detecting genuine homology (at least on synthetic data where this can be quantified). Here we have shown that the incorporation of an evolutionary model into Sigma's scoring scheme improves its sensitivity to the point where, on synthetic data, Sigma-2 is competitive with all other programs; while its precision is much higher, and error rate much lower, than all other programs that we tested.

Meanwhile, the motif-finding benchmark shows Sigma to be one of the best performers in a real-world application.

## Methods

### Evolutionary model

Stubb [20] and PhyloGibbs [2,3] use a model of evolution that differs in motivation from the Jukes-Cantor model and its descendants (including Felsenstein's model [12], which it resembles). Where Jukes-Cantor ask, "Given an observed rate of substitutions between two species, what is the evolutionary distance between them?", Stubb and PhyloGibbs ask "Given an evolutionary history that describes two or more organisms, and given a functional model that describes homologous loci in those organisms, what is the likelihood of the sequence observed at those loci?" The goal here was to distinguish between competing functional models (specifically, binding sites for transcription factors, statistically represented by "position weight matrices" [39,40]; and "non-functional", represented by a "background model".)

Calling the functional model *M*, let the probability of observing a nucleotide *α *at a particular locus be *M_α_*. Here, the vector of values *M_α _*could be a column of a position weight matrix, or the background probabilities of the four nucleotides, or something else. The assumption in the Stubb/PhyloGibbs model is that fixation operates sufficiently strongly that, if a site is mutated, it is also selected for, so that its distribution after mutation is again given by *M*. Suppose the nucleotide has descended from an ancestral nucleotide *β*, and the conservation rate or "proximity" (the probability of the nucleotide *not *having mutated) is *q*. The proximity is related to the mutation rate: if there are *μ *mutations in unit time, and the evolutionary time between the species is *t*, then *q *= exp(-*μt*). In is model, the "transition probability" (the probability of observing *α *given an ancestor *β*, the proximity *q*, and the model *M *is

(4) $$T(\alpha |\beta ;q,M)=q\delta_{\alpha \beta }+(1-q)M_{\alpha }.$$

In other words, with probability *q *the nucleotide is unmutated from the ancestor; and with probability 1 - *q *it is mutated (possibly multiple times), but also fixated, so that its distribution is given by *M*. If *μ *is the mutation rate, and the evolutionary time since the ancestor is *t*, then *q *= *e*^-*μt*^. This equation is the same as equation 7 in Felsenstein [12], with *M_α _*being his vector of equilibrium probabilities. The chief difference is that where Felsenstein had no functional model and his equilibrium probabilities were site-independent "background" probabilities, PhyloGibbs detects binding sites for transcription factors (TFs) as described by "position weight matrices" (PWMs), so *M_α _*is a single column of a PWM *W_nα_*, where *n *is the position within the putative binding sequence; thus the "equilibrium probabilities" are not site-independent, but--if a site is a TF binding site--are assumed to be precisely equal to the PWM that describes the binding of that TF. PhyloGibbs considered two possible functional models: binding sites for TFs, or background. Here we leave the model unspecified, but retain the assumption that the model describes the equilibrium probabilities.

This transition matrix has some desirable properties. It has reasonable limits as *q *→ 0 (zero conservation, where it reduces to *M*) and as *q *→ 1 (perfect conservation from the ancestor), and the correct composition with intermediate ancestors:

(5) $$\begin{array}{l} \sum_{\alpha =\text{A},\text{C},\text{G},\text{T}}T(\alpha |\beta ;q_{1},M)T(\gamma |\alpha ;q_{2},M)\\ =T(\gamma |\beta ;q_{1}q_{2},M). \end{array}$$

With PhyloGibbs, the model worked well, in the sense that the motif-finder based on it proved effective at finding regulatory sites in conserved sequence. However, it has some shortcomings that we address here.

First, the assumption of "perfect fixation" seems extreme in general, because restoring the original nucleotide requires at least two mutations at the same site--a doubly-rare event. (Felsenstein [12] is aware of this, but appears to define a "mutation" as a substitution of a nucleotide with any nucleotide, including possibly itself; he calls it a "useful compromise between realism and tractability.") Second, not all mutations are equally likely: transitions are much more common than transversions, and different transitions (and different transversions) occur at different rates, too.

The second shortcoming is easily addressed, and in doing so we move back in the direction of "standard" evolutionary models. Suppose that, if a mutation occurs, the probability of nucleotide *β *mutating to nucleotide *α *is given by the matrix *P_αβ_*. (The diagonal elements of this matrix are zero, since a nucleotide does not mutate into itself; and its columns sum to 1.) The probability of *β *changing to α after *k *mutations is given by the *k*'th power of this matrix. Given a mutation rate *μ*, the probability of *k *mutations in time *t *is given by the Poisson distribution

(6) $$P(k\;\text{mutations})=\frac{{\left(\mu t\right)}^{k}e^{-\mu t}}{k!}$$

and the transition probability, summed over all possible numbers of mutations, is

(7) T(α|β)=e−μtδβα+∑k=1∞[(μt)ke−μtk!×∑γ1,…,γk−2Pαγ1Pγ1γ2…Pγk−2β]=(eμt(P−I))αβ=(qI−P)αβ

where we have used the earlier definition of the proximity: *q *= exp(-*μt*).

This equation is widely used with a different derivation and a slightly different notation: usually *μt*(*P - I*) is defined as a single matrix that appears in a rate equation (eg, equation 2.7 in [10], or equation 3 in [11]).

To this framework we would like to add model-based selection and fixation. We make the assumption that *selection operates faster than mutation*: that is, while mutations are rare events at any given locus, the spread or disappearance of mutations at individual loci through a population occurs relatively rapidly. In that case, one can effectively replace *P *with an effective mutation probability matrix *P*' which includes the effect of fitness selection on mutations. So the probability of observing the nucleotide *α *at a particular locus, given that it has recently mutated from an ancestor *β*, depends (as above) on *β *and the mutation matrix *P*; but also by its function (described by a functional model *M*), for which it is being selected. Neighbour-dependence effects can in principle be absorbed into *M*: that is, the fitness of a nucleotide at a given position may depend on its neighbours. In this way, we incorporate correlated "background models" into our formalism. The neighbour-dependence of the background model is the only form of position specificity that we consider here, but in principle we can consider entirely different locus-specific functional models *M*, that describe transcription factor binding, nucleosome occupancy, or other features. We hope to extend Sigma in this manner in the future.

Given *M*, *β *and *P*, what is the probability of observing *α*? We use a Bayesian answer:

(8) $$P(\alpha |\beta ;M)=\frac{P(\beta |\alpha )P(\alpha |M)}{\sum_{\alpha^{\prime }}P(\beta |\alpha^{\prime })P(\alpha^{\prime }|M)}$$

Here *P*(*β*|*α*) is an "inverse mutation matrix", the probability that the ancestor was *β *given that the descendant is α. Defining $P_{\alpha \beta }^{\prime }=P(\alpha |\beta ;M)$, we have for our evolutionary model

(9) $$T(\alpha |\beta ;q,M)={\left(q^{I-P^{\prime }}\right)}_{\alpha \beta }.$$

**Figure 5:**
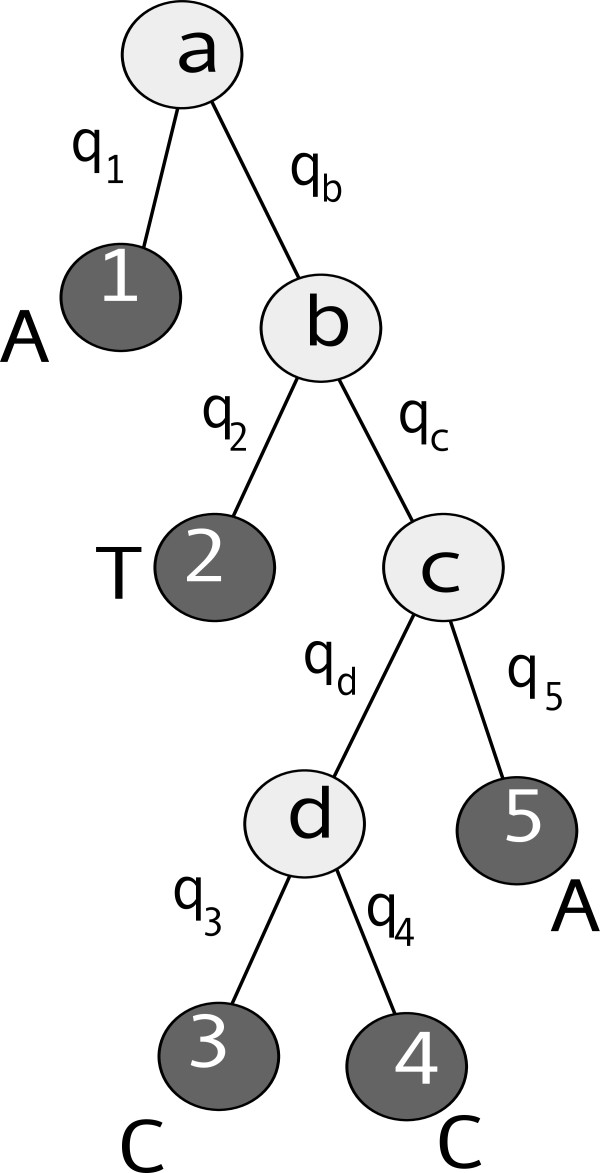
**An example of a tree for five species**. The branch lengths are proximities, the uppercase letters at the leaves are nucleotides at specific loci in the given sequences, and the lowercase letters in the nodes are unknown ancestral nucleotides at those loci. Transition probabilities are calculated as described in the main text.

The transition matrix allows us to evaluate the likelihood of a set of observed aligned nucleotides, given the phylogenetic tree that connects them and a functional model *M*. The likelihood is the product of transition probabilities over all branches of the tree, summed over the allowed nucleotides at the ancestor and at all intermediate nodes. For example, for a set of *N *descendants α*_i _*that all diverged from a single common ancestor *β *with proximities *q_i _*(*i *= 1 ...*N*), the likelihood is

(10) $$\begin{array}{l} P(\{\alpha_{i}\}|\beta ;q_{i},M)\\ =\sum_{\beta =\text{A},\text{C},\text{G},\text{T}}p_{\beta }\prod_{i=1}^{N}T(\alpha_{i}|\beta ;q_{i},M) \end{array}$$

and for the tree shown in Figure 5, the likelihood is

(11) $$\begin{array}{l} P({\text{A}}^{1}{\text{T}}^{2}{\text{C}}^{3}{\text{C}}^{4}{\text{A}}^{5}|T)=\\ \sum_{a}P_{a}T({\text{A}}^{1}|a,q_{1})\times \sum_{b}T(b|a,q_{b})T({\text{T}}^{2}|b,q_{2})\\ \times \sum_{c}T(c|b,q_{c})T({\text{A}}^{5}|c,q_{5})\\ \times \sum_{d}T(d|c,q_{d})T({\text{C}}^{3}|d,q_{3})T({\text{C}}^{4}|d,q_{4}). \end{array}$$

(Superscripts to nucleotides here indicate the leaves where they occur.)

We next describe how to apply these ideas to multiple sequence alignment.

### The sequence alignment algorithm

Sigma builds a global alignment progressively out of gapless local alignments. (Dialign-2 [22] previously used a similar strategy, but assembled the global alignment after evaluating all possible local alignments, rather than build it up progressively.) Local alignments are sorted according to their *p*-value, that is, the probability that an alignment with a similar or better similarity score would be found under the "null hypothesis" that the sequences are unrelated; and are made in increasing order of *p*-value. Alignments whose *p*-value is above a certain threshold, chosen by default to prevent alignment of random sequence, are rejected.

**Figure 6:**
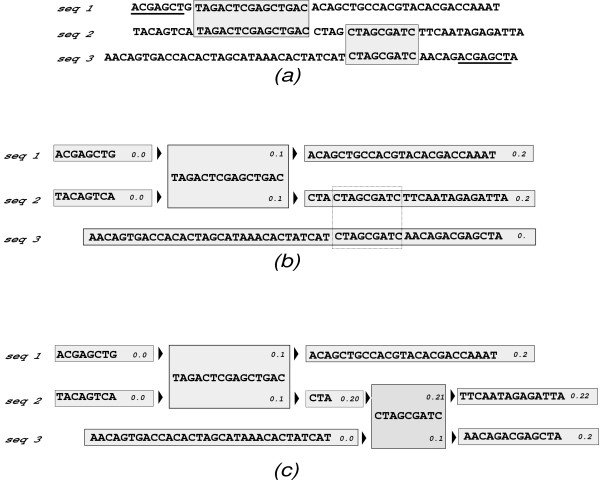
**Progressive alignment via "sequence fragments"**. Progressive alignment of input sequences. (a) Each sequence is initially its own fragment. All fragments are compared, pairwise, to find the best gapless local alignments. In this case, the grey boxes indicate two possible local alignments, and the underlined sequences show a third possible local alignment that is of lower significance, and also conicts with the first two. (b) The first local alignment is carried out by "fusing" two portions of seq1 and seq2 into one fragment. (c) The second local alignment is carried out, resulting in 9 sequence fragments at this point. However, the third local alignment, the underlined sequence in part (a), now conflicts with the existing alignments and cannot be performed. This is ensured by consistency conditions, enforced using a labelling scheme described in detail in [21] and illustrated here: each sequence in each fragment has a label (a string representation of a floating-point number) that is augmented whenever two fragments are "fused", in such a way that the numeric value of the label always increases from left to right along any sequence. Here, in (c), the fragment on seq3 labelled "0.2" can be aligned with the fragment on seq1 labelled "0.2", but not with any other fragment on seq1. (Figure reproduced from ref. [21])

The alignment strategy of Sigma is described in detail in the earlier paper [21]. Briefly, it works as follows: the basic data structure is a "sequence fragment". At any point in time, the alignment is given by a collection of "sequence fragments", corresponding to gapless local alignments of the input sequences. The input sequences are numbered, and initially every sequence is in its own fragment; as the multiple alignment progresses, each sequence fragment may contain multiple sequences (each numbered with the input sequence from which it originated), representing local *gapless *alignments. At each step, local alignments are made with all existing pairs of sequence fragments provided that the pairs are disjoint in the sequence numbers that they contain, and that aligning them would be consistent with previous alignments (synteny is preserved in alignments, and consistency is maintained via a labelling scheme). This is portrayed in Figure 6.

The local alignments are sorted by their *p*-values, and made in order of increasing *p*-value. Alignments whose *p*-value are greater than a given threshold are rejected. Each local alignment consists of "fusing" gapless stretches of two existing fragments into a single fragment containing the union of their nucleotides. The two aligned sequence fragments are replaced by five new fragments: the fused fragments, and two unfused fragments on either side (some or all of which may be of zero length).

So, initially, there are *N *fragments belonging to *N *input sequences (for example, 3 sequences in Figure 6). After a single round of local alignments, there is a larger number of fragments (for example, the 9 fragments in part (c) of Figure 6). After this, a new set of local alignments is calculated (with consistency conditions imposed) and performed on these fragments, which could result in a still larger number of fragments. This is repeated until there are no more possible local alignments whose *p*-value is below the minimal threshold. Finally, the "fragments" are "assembled" into the final alignment.

**Figure 7:**
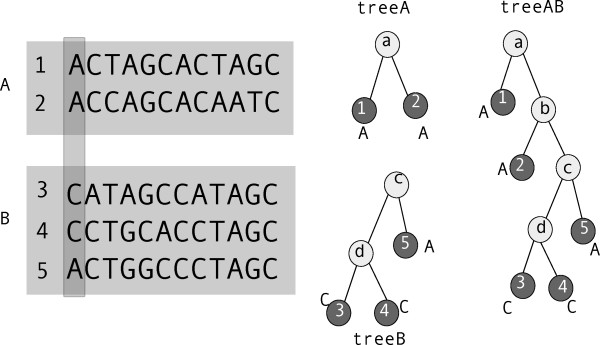
**Aligning two sequence fragments**. When two sequence fragments A and B, containing two and three sequences respectively, are aligned, the likelihood of the sequences if all five sequences are related (calculated from treeAB) is compared with the likelihood of those sequences if sequences 1 and 2 are unrelated to sequences 3, 4 and 5, but sequences within each fragment are related (treeA, treeB). The likelihoods, and log-likelihood ratio, are calculated as described in the main text.

The only major difference in algorithm with the previous program is in how the *p*-value is calculated. Sigma-1 calculated, in a rather crude way, the probability of seeing *m *mismatches in a local alignment of length ℓ, given total fragment lengths *L*_1 _and *L*_2_. In Sigma-2, as described in the "Evolutionary Model" subsection, the measure of the quality of a local alignment is the log of the ratio of the likelihood of nucleotides in that alignment arising from a single common ancestor, to the likelihood that nucleotides in each column in a single fragment are related (having been previously aligned) but the two fragments are unrelated. This is illustrated in Figure 7. Each possible gapless local alignment has a log-likelihood ratio *S *as a measure of its quality, which is the sum of such log-likelihood ratios over every "column" of nucleotides in the alignment. From *S *we calculate the *p*-value of the alignment using the central limit theorem: the total score *S *is a product over positions *i*, within the alignment, of individual scores *s_i_*. Before performing the alignment, therefore, the mean $\bar{s}$ and variance *σ *of *s *for 1000 individual pairs of positions selected randomly from the two fragments are calculated. If the fragments are unrelated, the expected mean log-likelihood-ratio of an alignment of length *m *would be $m\bar{s}$ and the expected variance would be $\sqrt{m}\sigma$ (from the central limit theorem, for sufficiently large m). The probability that the observed log-likelihood ratio observed in unrelated sequence fragments would be *S *or lower is

(12) $$p^{\prime }=\frac{1}{2}\text{erfc}\left(\frac{S-m\bar{s}}{\sigma \sqrt{2m}}\right)$$

where erfc *x *is the "complementary error function" of x: erfc $x=\frac{2}{\sqrt{\pi }}\int_{x}^{\infty }\exp\;(-x^{2})dx$. This applies to a particular local alignment of length *m*. But we want to consider all possible local alignments of length *m *in the given fragments. Suppose the fragments have lengths *L*_1 _and *L*_2_: the probability that *no *pairwise alignment exists, of length *m *and score *S *or lower, is

(13) $$\;\;\;P(\text{no alignment})={\left(1-p^{\prime }\right)}^{\left(L_{1}-m+1)(L_{2}-m+1\right)}$$

where the exponent is the number of possible ways subsequences of length *m *can be chosen from the two fragments. So the probability that *at least one *local alignment of length *m *and score *S *or lower exists in the two fragments, that is, our desired *p*-value, is

(14) $$\begin{array}{c} P=P(\text{at least one alignment})\\ =1-{\left(1-p^{\prime }\right)}^{\left(L_{1}-m+1\right)\left(L_{2}-m+1\right)}. \end{array}$$

For sufficiently small *p'*, this reduces to *p'*(*L*_1 _- *m *+ 1)(*L*_2 _- *m *+ 1). We note that Dialign-2 [41] uses a similar formula, *p'L*_1_*L*_2_, which amounts to assuming that *L*_1 _and *L*_2 _are large compared to *m*. However, *L*_1 _and *L*_2 _for Dialign-2 are the lengths of the original input sequences, while for us they are the lengths of the "sequence fragments" currently being considered under our "progressive alignment" scheme. If *L*_1 _and *L*_2 _are small (comparable in size to *m*), the significance increases: a small local alignment that would be rejected in the "first pass" may prove to be significant in context of local alignments that have previously been carried out. For example, if the original input sequences were each 1000 bp, a 10 bp local alignment may initially be insignificant; but if two large local alignments are carried out on either side of this 10 bp stretch, reducing the "available" sequence fragments to 50 bp each, the 10 bp local alignment may now become significant.

The best (lowest *p*) local alignment is found by a dynamic programming algorithm similar to the Smith-Waterman method [42]; but since the alignments are gapless, the algorithm requires only linear space but quadratic time, O(*L*_1_*L*_2_). Estimating the full running time of the program is less straightforward, since many local alignments are performed.

This algorithm requires a phylogenetic tree. Given input sequences, in a preliminary run Sigma-2 runs a multiple alignment with a "star phylogeny" tree where each sequence has a proximity of 0.33 from its ancestor. It uses this interim alignment to calculate all pairwise proximities. It then uses these pairwise values to construct a phylogenetic tree that is then used to perform the final alignment. While in principle this could be iterated to convergence, it seems to be unnecessary to do so.

## Availability

Sigma-2 is available from http://www.imsc.res.in/~rsidd/sigma2/ and is free software, distributable under the GNU General Public Licence.

## Authors' contributions

RS conceived the work. GJ and RS implemented the program and performed the benchmarks. RS primarily wrote the manuscript. Both authors read and approved the final manuscript.

## References

[B1] Padmanabhan S Thakur J Siddharthan R Sanyal K Rapid evolution of Cse4p-rich centromeric DNA sequences in closely related pathogenic yeasts, Candida albicans and Candida dubliniensis Proceedings of the National Academy of Sciences 2008 105 50 19797 19802 10.1073/pnas.0809770105 PMC2604992 19060206

[B2] Siddharthan R Siggia ED van Nimwegen E PhyloGibbs: A Gibbs Sampling Motif Finder That Incorporates Phylogeny PLoS Computational Biology 2005 1 7 e67 10.1371/journal.pcbi.0010067 16477324 PMC1309704

[B3] Siddharthan R PhyloGibbs-MP: Module Prediction and Discriminative Motif-Finding by Gibbs Sampling PLoS Comput Biol 2008 4 8 e1000156 18769735 10.1371/journal.pcbi.1000156 PMC2518514

[B4] Reeck GR de Haën C Teller DC Doolittle RF Fitch WM Dickerson RE Chambon P McLachlan AD Margoliash E Jukes TH "Homology" in proteins and nucleic acids: a terminology muddle and a way out of it Cell 1987 50 5 667 10.1016/0092-8674(87)90322-9 3621342

[B5] Jukes T Cantor C Evolution of protein molecules 1969 3 Academic Press, New York 21 132

[B6] Kimura M A simple method for estimating evolutionary rates of base substitutions through comparative studies of nucleotide sequences Journal of Molecular Evolution 1980 16 2 111 120 10.1007/BF01731581 7463489

[B7] Kimura M Estimation of evolutionary distances between homologous nucleotide sequences Proceedings of the National Academy of Sciences of the United States of America 1981 78 454 458 10.1073/pnas.78.1.454 6165991 PMC319072

[B8] Tamura K Estimation of the number of nucleotide substitutions when there are strong transition-transversion and G+C-content biases Mol Biol Evol 1992 9 4 678 687 1630306 10.1093/oxfordjournals.molbev.a040752

[B9] Tamura K Nei M Estimation of the number of nucleotide substitutions in the control region of mitochondrial DNA in humans and chimpanzees Mol Biol Evol 1993 10 3 512 526 8336541 10.1093/oxfordjournals.molbev.a040023

[B10] Tavaré S Some probabilistic and statistical problems in the analysis of DNA sequences 1986 17 American Mathematical Society 57 86

[B11] Rodríguez F Oliver JL Marín A Medina JR The general stochastic model of nucleotide substitution Journal of Theoretical Biology 1990 142 4 485 501 10.1016/S0022-5193(05)80104-3 2338834

[B12] Felsenstein J Evolutionary trees from DNA sequences: A maximum likelihood approach Journal of Molecular Evolution 1981 17 6 368 376 10.1007/BF01734359 7288891

[B13] Hasegawa M Kishino H aki Yano T Dating of the human-ape splitting by a molecular clock of mitochondrial DNA Journal of Molecular Evolution 1985 22 2 160 174 10.1007/BF02101694 3934395

[B14] Uzzell T Corbin KW Fitting discrete probability distributions to evolutionary events Science (New York, N.Y.) 1971 172 988 1089 1096 5574514 10.1126/science.172.3988.1089

[B15] Cooper DN Gerber-Huber S DNA methylation and CpG suppression Cell Differentiation 1985 17 3 199 205 10.1016/0045-6039(85)90488-9 3902251

[B16] Arndt PF Hwa T Identification and measurement of neighbor-dependent nucleotide substitution processes Bioinformatics 2005 21 10 2322 2328 10.1093/bioinformatics/bti376 15769841

[B17] Peng C Buldyrev SV Goldberger AL Havlin S Sciortino F Simons M Stanley HE Long-range correlations in nucleotide sequences Nature 1992 356 6365 168 170 10.1038/356168a0 1301010

[B18] Baele G de Peer YV Vansteelandt S A Model-Based Approach to Study Nearest-Neighbor Influences Reveals Complex Substitution Patterns in Non-coding Sequences Syst Biol 2008 57 5 675 692 10.1080/10635150802422324 18853356

[B19] Segal E Fondufe-Mittendorf Y Chen L Thastrom A Field Y Moore IK Wang JZ Widom J A genomic code for nucleosome positioning Nature 2006 442 7104 772 778 10.1038/nature04979 16862119 PMC2623244

[B20] Sinha S van Nimwegen E Siggia ED A probabilistic method to detect regulatory modules Bioinformatics 2003 19 suppl_1 i292 301 10.1093/bioinformatics/btg1040 12855472

[B21] Siddharthan R Sigma: multiple alignment of weakly-conserved non-coding DNA sequence BMC Bioinformatics 2006 7 143 10.1186/1471-2105-7-143 16542424 PMC1468434

[B22] Morgenstern B DIALIGN 2: improvement of the segment-to-segment approach to multiple sequence alignment Bioinformatics 1999 15 3 211 218 10.1093/bioinformatics/15.3.211 10222408

[B23] Thorne J Kishino H Felsenstein J An evolutionary model for maximum likelihood alignment of DNA sequences Journal of Molecular Evolution 1991 33 2 114 124 10.1007/BF02193625 1920447

[B24] Thorne JL Kishino H Felsenstein J Inching toward reality: An improved likelihood model of sequence evolution Journal of Molecular Evolution 1992 34 3 16 10.1007/BF00163848 1556741

[B25] Steel M Hein J Applying the Thorne-Kishino-Felsenstein model to sequence evolution on a star-shaped tree Applied Mathematics Letters 2001 14 679 684 10.1016/S0893-9659(01)80026-4

[B26] Subramanian A Kaufmann M Morgenstern B DIALIGN-TX: greedy and progressive approaches for segment-based multiple sequence alignment Algorithms for Molecular Biology 2008 3 6 10.1186/1748-7188-3-6 18505568 PMC2430965

[B27] Notredame C Higgins DG Heringa J T-coffee: a novel method for fast and accurate multiple sequence alignment Journal of Molecular Biology 2000 302 205 217 10.1006/jmbi.2000.4042 10964570

[B28] Thompson JD Higgins DG Gibson TJ CLUSTAL W: improving the sensitivity of progressive multiple sequence alignment through sequence weighting, position-specific gap penalties and weight matrix choice Nucl Acids Res 1994 22 22 4673 4680 10.1093/nar/22.22.4673 7984417 PMC308517

[B29] Larkin M Blackshields G Brown N Chenna R McGettigan P McWilliam H Valentin F Wallace I Wilm A Lopez R Thompson J Gibson T Higgins D Clustal W and Clustal X version 2.0 Bioinformatics 2007 23 21 2947 2948 10.1093/bioinformatics/btm404 17846036

[B30] Lassmann T Sonnhammer E Kalign - an accurate and fast multiple sequence alignment algorithm BMC Bioinformatics 2005 6 298 10.1186/1471-2105-6-298 16343337 PMC1325270

[B31] Brudno M Do CB Cooper GM Kim MF Davydov E Program NCS Green ED Sidow A Batzoglou S LAGAN and Multi-LAGAN: Efficient Tools for Large-Scale Multiple Alignment of Genomic DNA Genome Research 2003 13 4 721 731 10.1101/gr.926603 12654723 PMC430158

[B32] Edgar RC MUSCLE: multiple sequence alignment with high accuracy and high throughput Nucl Acids Res 2004 32 5 1792 1797 10.1093/nar/gkh340 15034147 PMC390337

[B33] Pei J Sadreyev R Grishin NV PCMA: fast and accurate multiple sequence alignment based on profile consistency Bioinformatics 2003 19 3 427 428 10.1093/bioinformatics/btg008 12584134

[B34] Bradley RK Roberts A Smoot M Juvekar S Do J Dewey C Holmes I Pachter L Fast Statistical Alignment PLoS Comput Biol 2009 5 5 e1000392 10.1371/journal.pcbi.1000392 19478997 PMC2684580

[B35] Paten B Herrero J Beal K Fitzgerald S Birney E Enredo and Pecan: Genome-wide mammalian consistency-based multiple alignment with paralogs Genome Research 2008 18 11 1814 1828 10.1101/gr.076554.108 18849524 PMC2577869

[B36] Bray N Pachter L MAVID: Constrained Ancestral Alignment of Multiple Sequences Genome Res 2004 14 4 693 699 10.1101/gr.1960404 15060012 PMC383315

[B37] Zhu J Zhang M SCPD: a promoter database of the yeast Saccharomyces cerevisiae Bioinformatics 1999 15 7 607 611 http://rulai.cshl.edu/SCPD/10.1093/bioinformatics/15.7.607 10487868

[B38] Kim J Sinha S Towards realistic benchmarks for multiple alignments of non-coding sequences BMC Bioinformatics 2010 11 54 10.1186/1471-2105-11-54 20102627 PMC2823711

[B39] Stormo GD Hartzell GW Identifying protein-binding sites from unaligned DNA fragments Proc Natl Acad Sci USA 1989 86 4 1183 1187 10.1073/pnas.86.4.1183 2919167 PMC286650

[B40] Hertz GZ Hartzell GW Stormo GD Identification of consensus patterns in unaligned DNA sequences known to be functionally related Comput Appl Biosci 1990 6 2 81 92 2193692 10.1093/bioinformatics/6.2.81

[B41] Morgenstern B Atchley W Hahn K Dress A Segment-based scores for pairwise and multiple sequence alignments Proceedings of the Sixth International Conference on Intelligent Systems for Molecular Biology 1998 AAAI Press, Menlo Park, CA 115 121 9783216

[B42] Smith TF Waterman MS Identification of common molecular subsequences Journal of Molecular Biology 1981 147 195 197 10.1016/0022-2836(81)90087-5 7265238

